# Chronic Lead Exposure and Mixed Factors of Gender×Age×Brain Regions Interactions on Dendrite Growth, Spine Maturity and NDR Kinase

**DOI:** 10.1371/journal.pone.0138112

**Published:** 2015-09-14

**Authors:** Yang Du, Meng-Meng Ge, Weizhen Xue, Qian-Qian Yang, Shuang Wang, Yi Xu, Hui-Li Wang

**Affiliations:** School of Biotechnology and Food Engineering, Hefei University of Technology, Hefei, Anhui 230009, P. R. China; Max-Delbrück Center for Molecular Medicine (MDC), GERMANY

## Abstract

NDR1/2 kinase is essential in dendrite morphology and spine formation, which is regulated by cellular Ca^2+^. Lead (Pb) is a potent blocker of L-type calcium channel and our recent work showed Pb exposure impairs dendritic spine outgrowth in hippocampal neurons in rats. But the sensitivity of Pb-induced spine maturity with mixed factors (gender×age×brain regions) remains unknown. This study aimed to systematically investigate the effect of Pb exposure on spine maturity in rat brain with three factors (gender×age×brain regions), as well as the NDR1/2 kinase expression. Sprague–Dawley rats were exposed to Pb from parturition to postnatal day 30, 60, 90, respectively. Golgi-Cox staining was used to examine spine maturity. Western blot assay was applied to measure protein expression and real-time fluorescence quantitative PCR assay was used to examine mRNA levels. The results showed chronic Pb exposure significantly decreased dendritic length and impaired spine maturity in both rat hippocampus and medial prefrontal cortex. The impairment of dendritic length induced by Pb exposure tended to adolescence > adulthood, hippocampus > medial prefrontal cortex and female > male. Pb exposure induced significant damage in spine maturity during adolescence and early adult while little damage during adult in male rat brain and female medial prefrontal cortex. Besides, there was sustained impairment from adolescence to adulthood in female hippocampus. Interestingly, impairment of spine maturity followed by Pb exposure was correlated with NDR1/2 kinase. The reduction of NDR1/2 kinase protein expression after Pb exposure was similar to the result of spine maturity. In addition, *NDR2* and their substrate *Rabin3* mRNA levels were significantly decreased by Pb exposure in developmental rat brain. Taken together, Pb exposure impaired dendrite growth and maturity which was subject to gender×age×brain regions effects and related to NDR1/2 signal expression.

## Introduction

Lead (Pb) is an important metal pollutant in not only natural environment but also transportation and food processing industry. Pb is very easy to be uptaked into organisms and developed to be bioaccumulated, further engendering biological toxic effects, like neurodevelopmental and other physical developmental toxic effects [1–5]. The toxicity induced by Pb exposure is affected by many factors, like different developmental phase, sex and so on. A large number of studies have shown that developmental Pb exposure can induce impairment of cognitive function, learning and memory [6–9].) In addition, some studies give scientific evidence that gender also plays another important role in Pb accumulation [10, 11] and cognitive function [12–16].

Brain is full of networks to maintain its complex function and usually several brain regions interaction supports one function. Recent study [17] reported the functional connectivity in cortical-hippocampal network and then the cooperative function in improving associative memory. It offered a sight of the brain function between brain regions. Previous studies have shown Pb exposure induced impairment in hippocampus [16, 18], as well as cerebral cortex [19], respectively. So what is the alternation between these two brain regions in response to Pb-exposed neurotoxicity? Is cooperative or unrelated?

Dendrite is one or more protrusions originate from cell body, with a function of receiving stimulation and transmitting impulse to somas. There are spines distribute along dendrites, which is called as dendritic spine. Dendritic spine is closely related to synaptic strength, which is regulated by Ca^2+^ level [20, 21]. Previous studies showed that Pb affected dendritic spine formation in rat brain [18]. In addition, Pb blocks voltage dependent calcium channel and induces long term potentiation (LTP) dysfunction [22–24]. So present study regarded dendritic length and mushroom spine as cognition marker to investigate the neurotoxicity followed by Pb exposure.

In regard to regulation of spine formation, multiple molecules involved in this process, including Brain-derived Neurotrophic Factor (BDNF) [25], Wnt related proteins [26], NDR (nuclear Dbf2-related) 1/2 kinase [27], Shank and Homer [28]. Among these proteins, NDR1/2 kinase drew our attention because of its regulation of the dynamic change in dendritic spine morphology and its key role in regulating dendrite growth [27]. It has been demonstrated that NDR plays an essential role in dendrite maintenance[29]. Other studies have shown NDR and its substrates were significant for regulating the processes of dendrite growth, dendrite branch, spine distribution, spine morphologies and so on [27]. Rabin 8, which is one of the substrates of NDR1/2, participates in regulating dendrite growth, especially spine growth [27]. NDR1/2 kinase family, a kind of NDR protein-kinases, is highly homologous from yeast to humans, and is responsible for many important cellular processes [30]. This kinase was expressed in mice brain and human beings tissues broadly [31, 32].Previous studies have shown NDR1/2 kinase could be activated by calmodulin s100. It was discovered that Ca^2+^, an endocellular second messager, could regulate the activity of NDR [33]. That indicated NDR probably involve in cellular signal transduction pathways.

In this study, we attempted to investigate the effects of chronic Pb exposure on dendritic length and mushroom spine formation with mixed factors of different genders (male, female), different ages (PND30, PND60, PND90) and different regions of the brain (hippocampus, medial prefrontal cortex). In addition, we explored NDR kinase protein expression, as well as the *NDR1*, *NDR2* and its substrate *Rabin8* transcription mRNA levels

## Materials and Methods

### Chronic Pb exposure animal model

Sprague–Dawley (SD) rats were obtained from the Laboratory Animal Center, Anhui Medical University, P.R. China. Animals were individually housed in a temperature and humidity controlled environment on a 12h–12h light–dark cycle with free access to food and water. All experimental operations were performed following the guidelines of the National Institute of Health Guide for the Care and Use of Laboratory Animals and were approved by the Institutional Animal Care and Use Committee of Hefei University of Technology, China.

**Fig 1:**
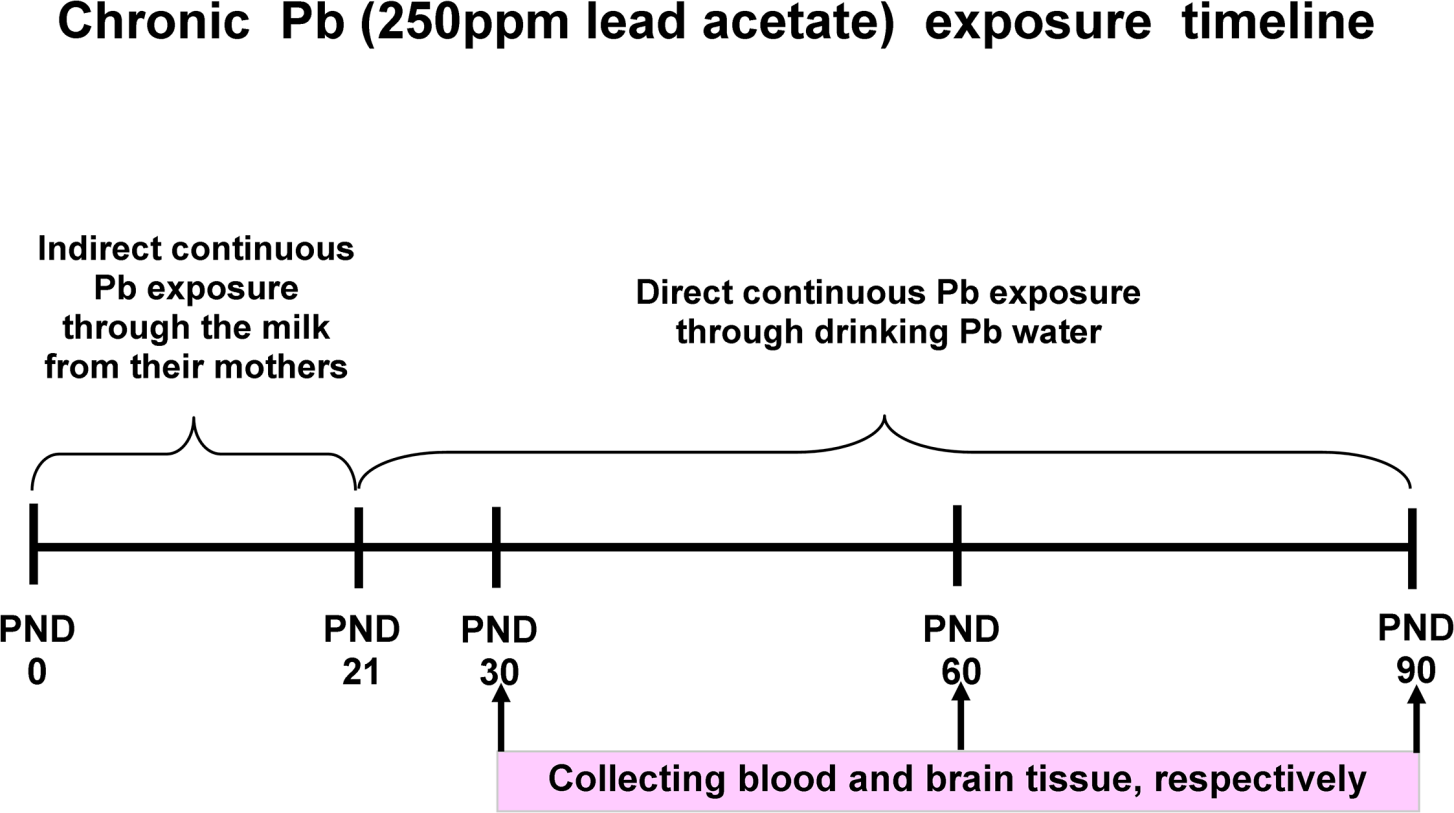
Illustration of the overall research design timeline.

The method for chronic Pb exposure was referred to the previous study [34]. Female SD rats were raised individually after mating and treated with distilled water before childbirth, and then the rats were exposed to Pb or not. SD rat dams were randomly divided into 12 groups: drinking distilled water as control (male, female) at PND30, PND60, PND90, and Pb water (250 ppm lead acetate in distilled water, 30 ml/day) as Pb-exposed group (male, female) at PND30, PND60, PND90, respectively (n = 8 per group). The Pb-exposed rat pups acquired Pb during lactation indirectly through the milk from their mothers and then directly after weaning at the postnatal day 21 (PND21) until they were decapitated (PND30, PND60 and PND90, respectively). The rats were monitored every day after treatment and were normal in activity and diet. There was no unintended death of animals during this study. Fig 1 illustrates the overall research design timeline.

### Tissue collection

SD rat pups and rats were anesthetized deeply with Carbon dioxide and then decapitated, then the brain was removed from the skull quickly [35]. In this study, SD rats were sacrificed at PND30, PND60 and PND90 after chronic Pb exposure, respectively. Brains were cut into two hemispheres longitudinally; the right part was prepared for morphological staining, and the left one for examining special proteins expression and real time fluorescence quantitative PCR assay.

### Blood and brain Pb levels examination

Tissue Pb level assay was performed as follows: brain tissue was added with nitric acid (excellent pure GR, 4 mL) and 30% hydrogen peroxide (AR, 2 mL) in the nitrolysistube overnight at room temperature. And then, those tissues were nitrolyzed for 30 min in the microwave nitratepyrolysis furnace (EMR marsxpresscertificate, VB 20). Lastly, the Pb level within sample was detected by the graphite furnace atomic spectrophotometry (the PerkinElmer AAnalyst^TM^800, USA).

Blood Pb level assay was performed as follows: 0.5 mL of blood was added into 4.5 mL dilution liquid (0.2% nitricacid and 0.1% TritonX-100). Each sample was vortexed for 2 min and the Pb level within sample was detected by the graphitefurnace atomic spectrophotometry (the PerkinElmer AAnalyst^TM^800, USA).

### Golgi-Cox staining, dendritic length and the number of dendritic spines assay

The brain was processed by Golgi-cox staining method as described by Liu et al [36]. Briefly speaking, the brains were stored in GC solution (37°C) in dark for 36–48 hours. Making brain slices at the thickness of 200μm with a vibratome (VT1000S, Leica, Germany), fixing them on 2% gelatin-coated slides. Five brain slices were collected from each rat. Then all slices were stained with following steps: with ammonia for 60 min, water for 3 times, Kodak Film Fix for 30min, after that, with water, dehydrated, cleared, and finally, mounted using a resinous medium. The neurons in hippocampus and medial prefrontal cortex (mPFC) were imaged with a Nikon widefield microscope (Eclipse 80i) by using a 10x&100x objectives. For all collected sections, 3~5 neurons were used of each slice with 10x&100x respectively.

Then dendritic length and the number of mushroom spines within those neurons were analyzed by Matlab software. In brief, dendrite morphology was analyzed across the concentric 10μm circles. The dendritic length counted in present study was of the first dendrite, and the mushroom spines were on 2~3 stretches of the secondary dendrite about 10 μm in length.

### Western Blotting assay

The tissue hippocampus was collected from rats at PND30, PND60 and PND90, respectively. The hippocampal protein was obtained by being homogenized in ice-cold lysis buffer (pH7.4; containing 21μg/ml aprotinin, 0.5μg/ml leupetin, 4.9mM MgCl_2_, 1mM sodium-Meta-vanandante, 1% Triton X-100 and 1mM PMSF), then centrifugated (14000×g, 7 min), then the supernatant was collected.

The total protein concentration was assayed by the Bicinchoninic Acid (BCA) method. And the equal quality protein of every sample was separated by 8% SDS-PAGE gel before transferring to PVDF membrane. Membranes were blocked with 5% non-fat dry milk, incubated with primary antibodies (GAPDH was purchased from Abcam, ab9484, monoclonal, 1:5000, NDR1/2 was from Santa cruz, sc271703, monoclonal, 1:2000), then washed for 3 times, incubated with the horseradish peroxidase-conjugated secondary antibody and processed for visualization by the enhanced chemiluminescence immuno-blotting detection system. All results were normalized against GAPDH.

### Real-time fluorescence quantitative PCR

The total RNAs in developmental hippocampus and mPFC were extracted using the RNA kit (Axygen, Silicon Valley, USA) from the hippocampus and mPFC of rats (*n* = 8 per group) with or without Pb exposure. Subsequently, the primer OligodT were used to complete the reverse transcription reaction according to the manufacturer's instructions (TransGene, Shanghai, China), resulting in the first strand of total cDNA.

The 20 μL reaction pool of RTFQ PCR was composed of: 10 μL of SYBR premix Extaq; 0.8 μL of forward and reverse primer each; 2 μL of cDNA template (10 times dilution) and 6.4 μL of deionized water. The primers used in this protocol were listed as follows: GGGTTAAGGGTGATTGATGTTCG-AGGCACCTCTATCTCCTTCGCA for *NDR1*, AGACGGAGCCTGGGTAGTGA- AAAGGTTGTCTGGCTTGATGTC for *NDR2*, GTTCCAGAGCCAGCATCATCG- TCATCGTTGCCAGCAGAAGC for *Rabin3* (*Rabin3*, a rat protein homologous to human protein *Rabin8*.) and CTGTGCTATGTTGCCCTAGACTTC-CATTGCCGATAGTGATGACCTG for *r-Actin*, respectively. The real-time fluorescence PCR system was purchased from Roche (Roche Lightcycler 96). The reaction procedure was set as one cycle of 95°C for 10 s, 40 cycles of 95°C 10 s, 60°C 30 s, followed by the melting stage of 95°C 10 s, 65°C 60 s and 97°C 1 s, then the cooling stage of 37°C 30s. The transcription levels were calculated as the amounts relative to that of *r-Actin* under the same conditions.

### Statistical analysis

All data is presented as means ± SEM. The statistical differences between groups were analyzed by Two-way (with factors of age & brain regions) or Three-way (gender×age×brain regions) ANOVA followed by a Fisher’s LSD-hoc test. A value of p<0.05 was considered as the statistical difference.

## Results

### Blood Pb level and brain Pb level

**Fig 2:**
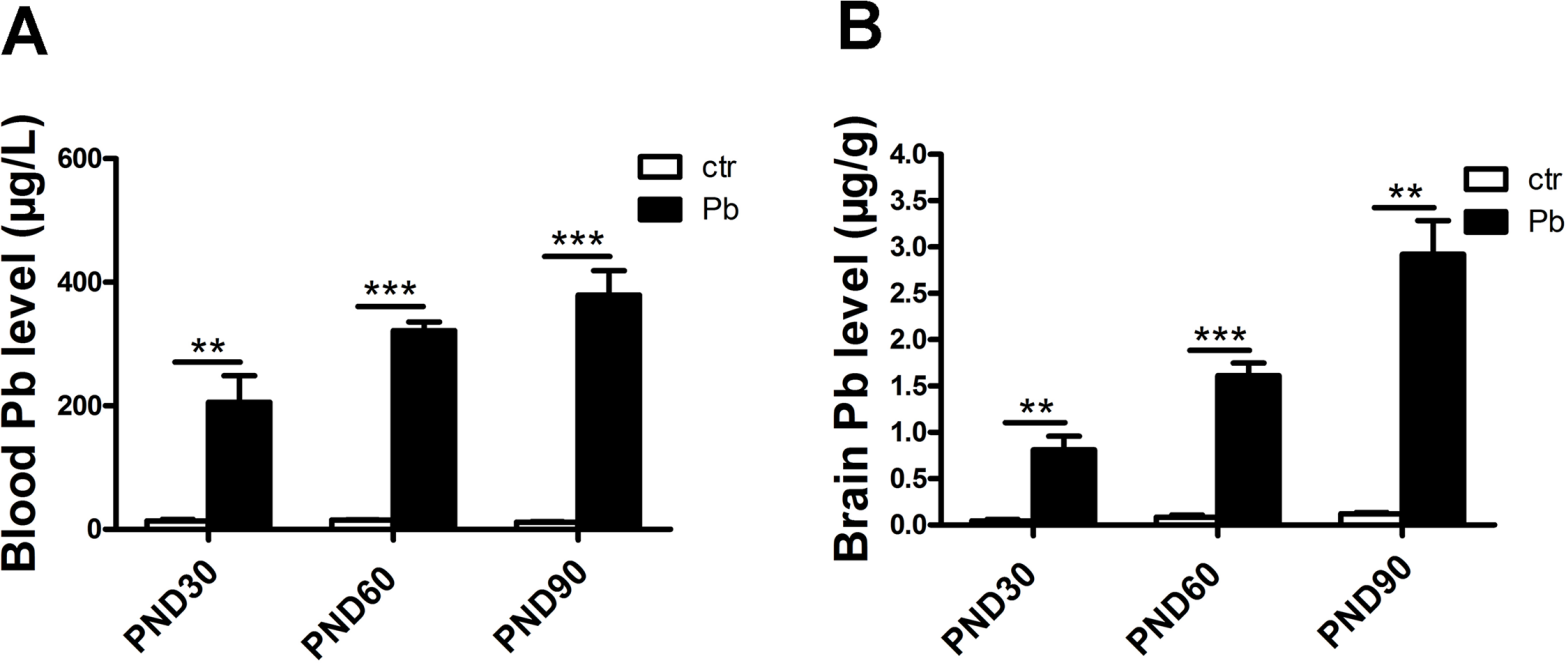
Pb accumulation in blood and brain in both control and Pb-exposed rats. Blood Pb levels in control and Pb-exposed rats at PND30, PND60 and PND90 (A). Brain Pb levels in control and Pb-exposed rats at PND30, PND60 and PND90 (B). Data are expressed as mean ± SEM. **P<0.01, ***P<0.001, n = 8 per group.

Pb accumulation within blood and brain was determined by the graphite furnace atomic spectrophotometry. Fig 2A showed blood Pb levels at PND30 (control, 13.958±2.188 μg/L; Pb exposure, 205.575±43.234 μg/L, F(1,6) = 19.593, P<0.01, n = 8), PND60 (control, 15.034±0.773 μg/L; Pb exposure, 321.963±13.691 μg/L, F(1,4) = 500.975, P<0.001, n = 8) and PND90 (control, 11.845±1.068 μg/L; Pb exposure, 379.167±39.435 μg/L, F(1,4) = 86.697, P<0.001, n = 8), respectively. Fig 2B showed brain Pb levels at PND30 (control, 0.046±0.015 μg/g; Pb exposure, 0.812±0.147 μg/g, F(1,6) = 26.832, P<0.01, n = 8), PND60 (control, 0.085±0.024 μg/g; Pb exposure, 1.611±0.137 μg/g, F(1,4) = 121.036, P<0.001, n = 8) and PND90(control, 0.122±0.0122 μg/g; Pb exposure, 2.921±0.365 μg/g, F(1,4) = 58.576, P<0.01, n = 8), respectively. Therefore, in either brain or blood, Pb levels in Pb-exposed rats significantly higher than those of the controls.

### The change of dendritic length in hippocampus and medial prefrontal cortex (mPFC) at different ages in Pb-exposed rats

**Fig 3:**
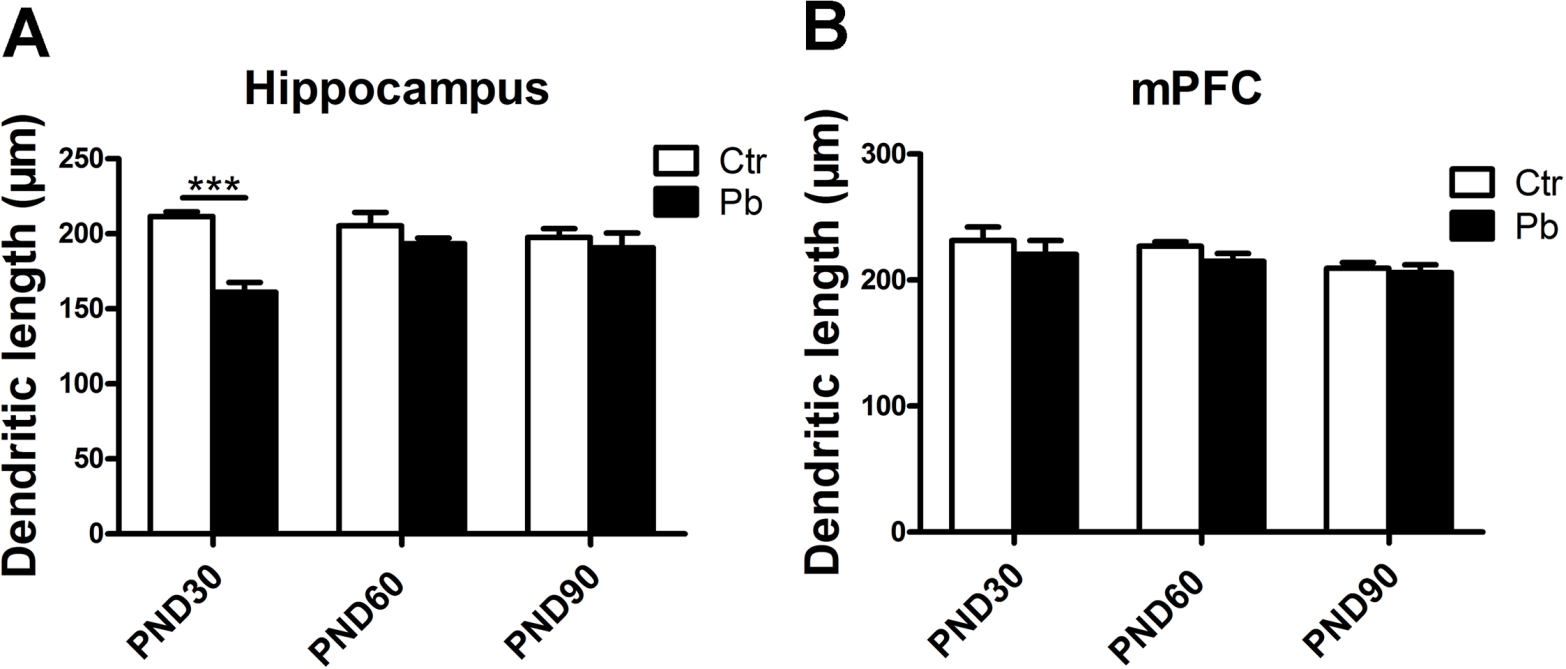
Effect of chronic Pb exposure on dendritic length in rat brain was subject to brain regions differences and age differences. Dendritic length in hippocampal neurons at PND30, PND60, PND90, respectively (A). Dendritic length in mPFC at PND30, PND60, PND90, respectively (B). Data are expressed as mean ± SEM. ***P<0.001. n = 16 per group.

Fig 3 showed that chronic Pb exposure decreased dendritic length in both hippocampus and mPFC at all three age (PND30, PND60 and PND90). Age-dependent differences were important in impairment caused by metal in rat brain regardless of sex [15]. The dendritic length in hippocampus was 211.578±3.063 μm, 205.373±8.865 μm, 197.537±5.841 μm in control group and 161.133±6.385 μm, 193.348±3.940 μm, 190.634±9.948 μm in Pb-exposed group at PND30, PND60 and PND90, respectively (Fig 3A). Significant decrease was observed in dendritic length in Pb-exposed group compared to control group at PND30 (F(1,72) = 65.308, P<0.001, n = 16), while the decrease was not significant in either PND60 (F(1,89) = 1.990, P>0.05, n = 16) or PND90 (F(1,47) = 0.365, P>0.05, n = 16) groups (Fig 3A). No significant difference was observed in dendritic length in Pb-exposed group compared to control group at PND30 (control, 231.354±10.810 μm; Pb exposure, 220.457±10.859 μm, F(1,63) = 0.410, P>0.05, n = 16), PND60 (control, 226.910±3.515 μm; Pb exposure, 214.945±6.112 μm, F(1,68) = 3.268, P>0.05, n = 16) and PND90 (control, 209.324±4.487 μm; Pb exposure, 205.783±6.222 μm, F(1,78) = 0.223, P>0.05, n = 16) in mPFC (Fig 3B).

These results suggested that the impairment of dendritic length after Pb exposure was mainly in developmental hippocampus in rat brain.

### Effects of gender differences on the alternation of dendritic length in chronic Pb-exposed rat brain

**Fig 4:**
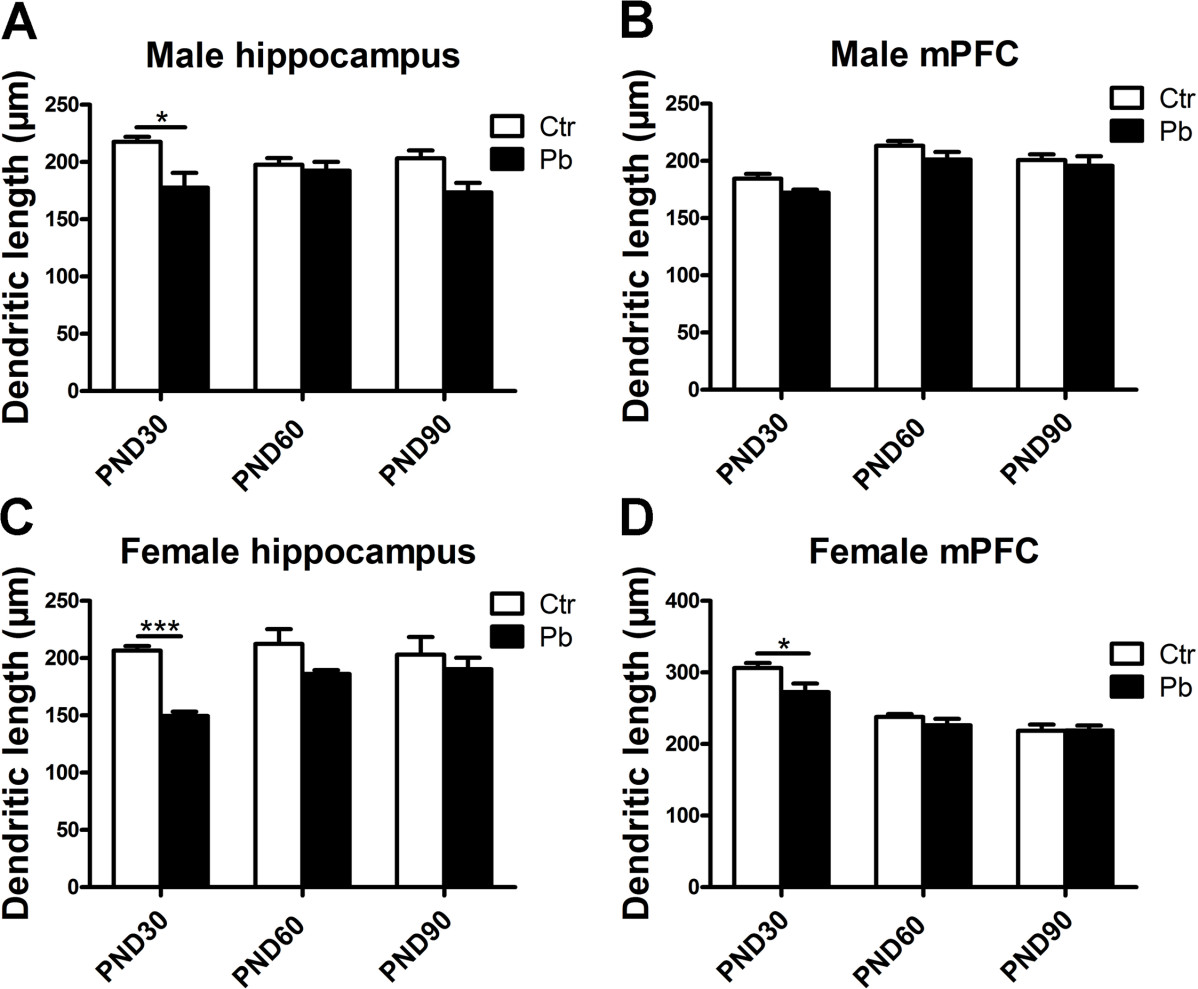
Effect of chronic Pb exposure on dendritic length in rat brain was subject to brain regions×age×gender age interaction. Dendritic length in male hippocampal neurons at PND30, PND60, PND90, respectively (A). Dendritic length in male mPFC at PND30, PND60, PND90, respectively (B). Dendritic length in female hippocampal neurons at PND30, PND60, PND90, respectively (C). Dendritic length in female mPFC at PND30, PND60, PND90, respectively (D). Data are expressed as mean ± SEM. *P<0.05, ***P<0.001. n = 8 per group.

Gender differences were also important in neurotoxicity caused by multiple metals [11, 37]. On the basis of the decrease in dendritic length after Pb exposure (Fig 3), we asked whether this impairment was subject to gender differences. As data shown in Fig 4, there was a decrease in dendritic length following Pb exposure in both two brain regions from PND30 to PND90. This decrease was significant in male hippocampus at PND30 (control, 217.508±4.574 μm; Pb exposure, 177.573±12.968 μm, F(1,31) = 13.396, P<0.05, n = 8) (Fig 4A), while the difference in male hippocampus at PND60 (control, 197.603±5.811 μm, Pb exposure, 192.367±7.752 μm, F(1,46) = 0.225, P>0.05, n = 8) and PND90 (control, 203.293±6.713 μm; Pb exposure, 173.369±8.406 μm, F(1,22) = 7.891, P>0.05, n = 8) was not (Fig 4A). Similar results were observed in female hippocampus, the decrease in dendritic length appeared to be more pronounced in Pb-exposed group at PND30 (control, 206.526±3.941 μm; Pb exposure, 149.390±3.878 μm, F(1,39) = 86.047, P<0.001, n = 8) while the data at PND60 (control, 212.468±12.918 μm; Pb exposure, 186.053±3.524 μm, F(1,41) = 3.734, P>0.05, n = 8) and PND90 (control, 202.966±15.399 μm; Pb exposure, 190.212±10.096 μm, F(1,23) = 0.424, P>0.05, n = 8) was not (Fig 4C).

Although no significant difference was observed in mPFC regardless of sex (Fig 3B), data in Fig 4B and 4D exhibited gender differences during adolescence. In male mPFC, there was no significant effect on dendritic length at PND30 (control, 184.493±4.156 μm; Pb exposure, 172.198±2.777 μm, F(1,35) = 6.417, P>0.05, n = 8), PND60 (control, 213.145±4.237 μm; Pb exposure, 201.149±6.592 μm, F(1,29) = 2.566, P>0.05, n = 8) and PND90 (control, 200.783±4.910 μm; Pb exposure, 195.698±8.300 μm, F(1,41) = 0.296, P>0.05, n = 8) after Pb exposure (Fig 4B). As shown in Fig 4D, a striking reduction was observed in dendritic length in Pb exposure group compared to control group at PND30 (control, 306.076±6.992 μm; Pb exposure, 272.405±12.178 μm, F(1,26) = 6.571, P<0.05, n = 8), a reduction trend but not statistically significant in PND60 group (control, 237.683±4.144 μm; Pb exposure, 226.155±8.9147 μm, F(1,37) = 1.671, P>0.05, n = 8) and almost no effect at the age of 90 days (control, 218.389±8.665 μm; Pb exposure, 218.679±7.300 μm, F(1,35) = 0.001, P>0.05, n = 8) in female mPFC (Fig 4D).

These results suggested that chronic Pb exposure decreased the dendritic length in both genders and this decrease was mainly during adolescence. In addition, difference was observed about the effect on dendritic length in developmental mPFC between genders.

### The alternation of mushroom spines in hippocampus and mPFC at different ages in Pb-exposed rats

**Fig 5:**
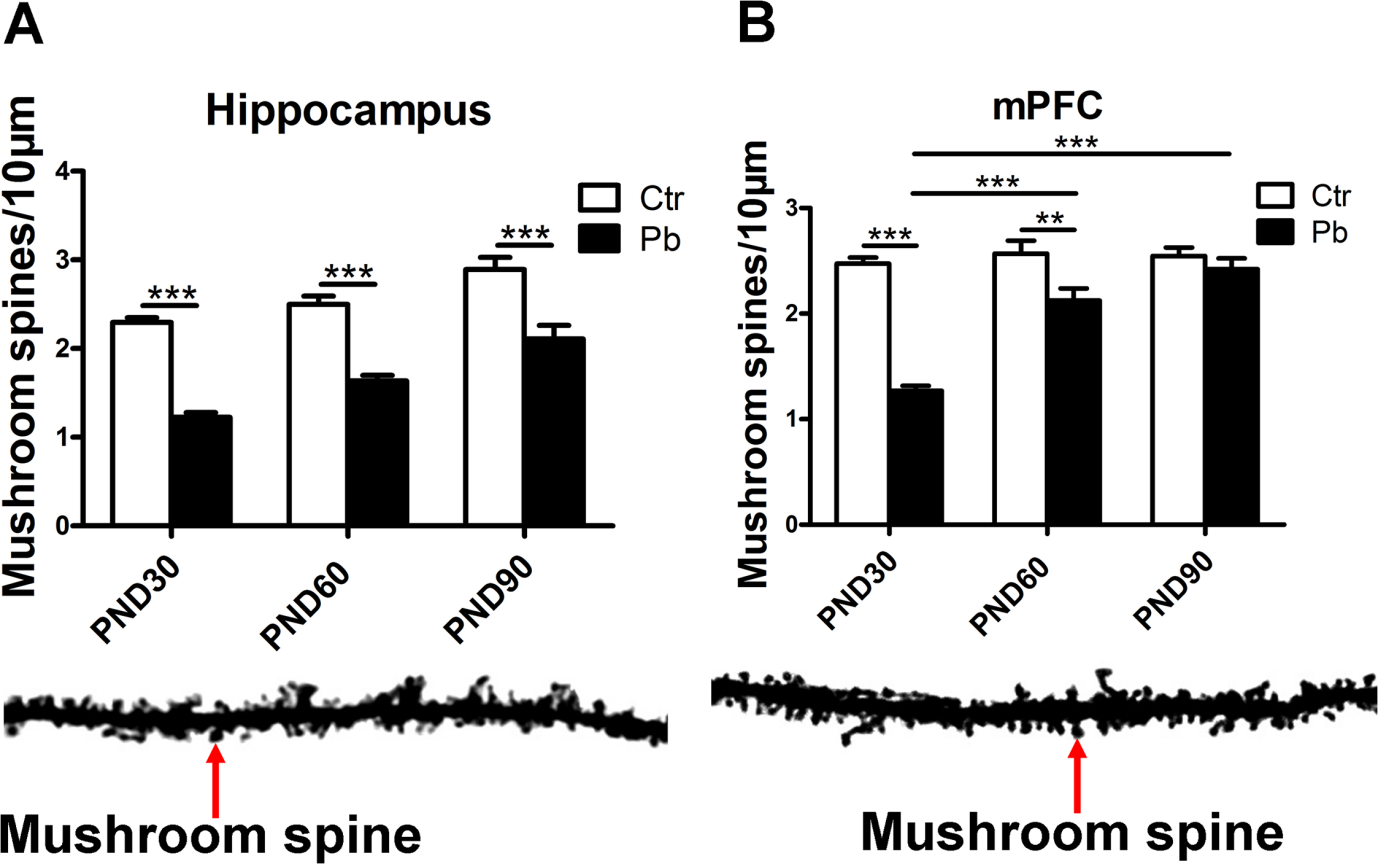
Effect of chronic Pb exposure on dendritic mushroom spines in rat brain with brain regions differences and age differences. The number of mushroom spines in hippocampal neurons at PND30, PND60, PND90, respectively (A). The number of mushroom spines in mPFC at PND30, PND60, PND90, respectively (B). Blow the histogram: dendritic spines stained with the Golgi-cox (Scale bar = 10μm). Data are expressed as mean ± SEM. **P<0.01, ***P<0.001. Scale bar = 10μm. n = 16 per group.

In view of the importance of mushroom spine in synapse [38, 39], we then explored whether the number of mushroom spines (/10μm) were subject to brain regions (hippocampus and mPFC) ×age (PND30, PND60 and PND90) effects. As shown in Fig 5A, Pb exposure significantly decreased the number of mushroom spines in hippocampus at PND30 (control, 2.295±0.052; Pb exposure, 1.226±0.051, F(1,141) = 189.075, P<0.001, n = 16), PND60 (control, 2.497±0.094; Pb exposure, 1.635±0.062, F(1,151) = 63.149, P<0.001, n = 16) and PND90 (control, 2.890±0.137; Pb exposure, 2.108±0.152, F(1,67) = 14.065, P<0.001, n = 16).

Chronic Pb exposure also significantly impaired mushroom spine formation in mPFC in adolescence (PND30) (control, 2.474±0.056; Pb exposure, 1.269±0.047, F(1,157) = 247.451, P<0.001, n = 16), early adulthood (PND60) (control, 2.569±0.122; Pb exposure, 2.124±0.115, F(1,132) = 6.834, P<0.01, n = 16) and did not induce considerable decrease at adulthood (PND90) (control, 2.547±0.078; Pb exposure, 2.420±0.102, F(1,100) = 0.998, P>0.05, n = 16) (Fig 5B). Additionally, an increase was observed about the number of mushroom spines in Pb-exposed groups from PND30 group to PND90 group when the corresponding control groups’ value was almost equal, and the increase was notable between PND60 and PND30 (P<0.001), between PND90 and PND30 (P<0.001) (Fig 5B). It indicated that the degree of impairment of spine maturity wore off from adolescence to adult in mPFC after chronic Pb exposure.

In summary, Pb exposure induced impairment in mushroom spine formation in both mPFC and hippocampus from developmental phase to adulthood, and this impairment was age-dependent in mPFC.

### Effects of gender on the number of mushroom spines in response to chronic Pb exposure in rat brain

**Fig 6:**
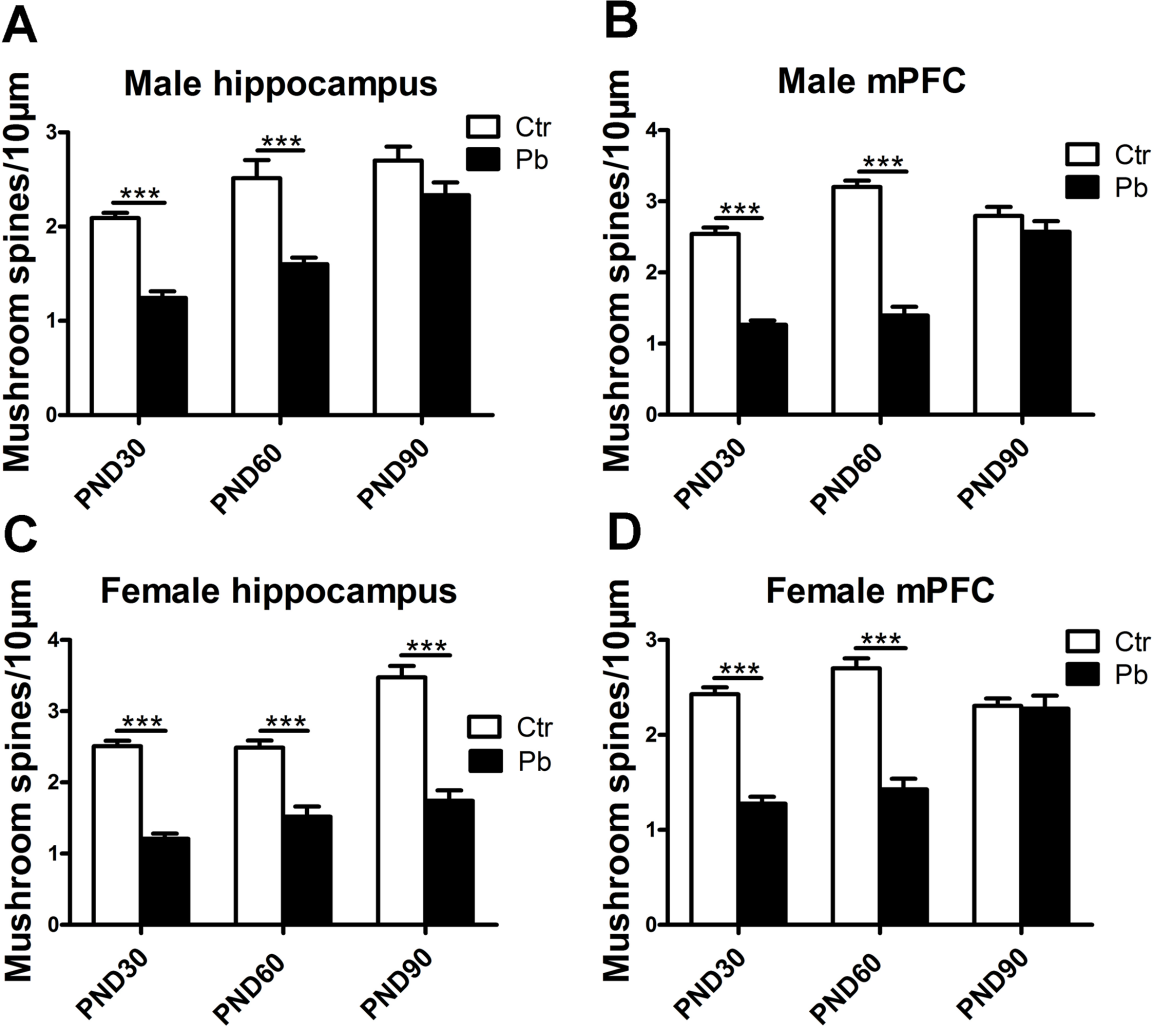
Effect of chronic Pb exposure on mushroom spines in rat brain was subject to brain regions×age×gender age interaction. The number of mushroom spines in male hippocampal neurons at PND30, PND60, PND90, respectively (A). The number of mushroom spines in male mPFC at PND30, PND60, PND90, respectively (B). The number of mushroom spines in female hippocampal neurons at PND30, PND60, PND90, respectively (C). The number of mushroom spines in female mPFC at PND30, PND60, PND90, respectively (D). Data are expressed as mean ± SEM. ***P<0.001. n = 8 per group.

Then we asked whether gender caused differences in mushroom spine formation in hippocampus and mPFC at different ages in Pb-exposed rats. Fig 6A showed chronic Pb exposure impaired mushroom spine formation in male hippocampus at PND30 (control, 2.091±0.055; Pb exposure, 1.244±0.070, F(1,72) = 90.591, P<0.001, n = 8), PND60 (control, 2.513±0.193; Pb exposure, 1.602±0.068, F(1,88) = 31.661, P<0.001, n = 8) and PND90 (control, 2.700±0.148; Pb exposure, 2.333±0.136, F(1,33) = 3.167, P>0.05, n = 8). In female hippocampus, significant impairment was observed about spine maturity in female hippocampus at PND30 (control, 2.509±0.077; Pb exposure, 1.207±0.075, F(1,67) = 124.630, P<0.001, n = 8), PND60 (control, 2.488±0.101; Pb exposure, 1.517±0.143, F(1,54) = 24.967, P<0.001, n = 8) and PND90 (control, 3.475±0.161; Pb exposure, 1.742±0.145, F(1,39) = 64.321, P<0.001, n = 8) (Fig 6C). As shown in Fig 6A and 6C, there was difference in hippocampal spine maturity at PND90 between genders.

In male mPFC, the number of mushroom spines was significantly lower in Pb exposure group compared to control at PND30 (control, 2.541±0.091; Pb exposure, 1.264±0.062, F(1,72) = 134.233, P<0.001, n = 8), PND60 (control, 3.201±0.091; Pb exposure, 1.395±0.122, F(1,72) = 142.345, P<0.001, n = 8) except for PND90 (control, 2.796±0.124; Pb exposure, 2.571±0.149, F(1,48) = 1.335, P>0.05, n = 8) (Fig 6B). Similar results were observed in female mPFC that the number of mushroom spines was significantly lower in Pb exposure group compared to control at PND30 (control, 2.429±0.071; Pb exposure, 1.276±0.072, F(1,83) = 111.975, P<0.001, n = 8) and PND60 (control, 2.702±0.104; Pb exposure, 1.426±0.113, F(1,58) = 68.306, P<0.001, n = 8) (Fig 6D), whereas there was little difference at PND90 (control, 2.306±0.076; Pb exposure, 2.277±0.136, F(1,50) = 0.041, P>0.05, n = 8) (Fig 6D).

Our results showed that Pb exposure impaired mushroom spine formation in both genders. Furthermore, this impairment was subject to gender differences in hippocampus between male and female rats during adulthood. It indicated Pb exposure induced enduring impairment on spine maturity in female hippocampus from adolescence to adulthood while male was just from adolescence to early adult.

### The alternation of NDR1/2 signaling molecules with the interaction of gender×age×brain regions in rat brain following chronic Pb exposure

It has been demonstrated that Pb affected cellular Ca^2+^ flux [23]. NDR 1/2 kinase protein, which might be regulated by endocellular second messager Ca^2+^, played an essential role in dendrite morphology and spine formation [33]. We then asked whether this dendrite-related kinase was affected by Pb exposure and whether the alternation of the expression induced by Pb was subject to the above-mentioned three factors.

**Fig 7:**
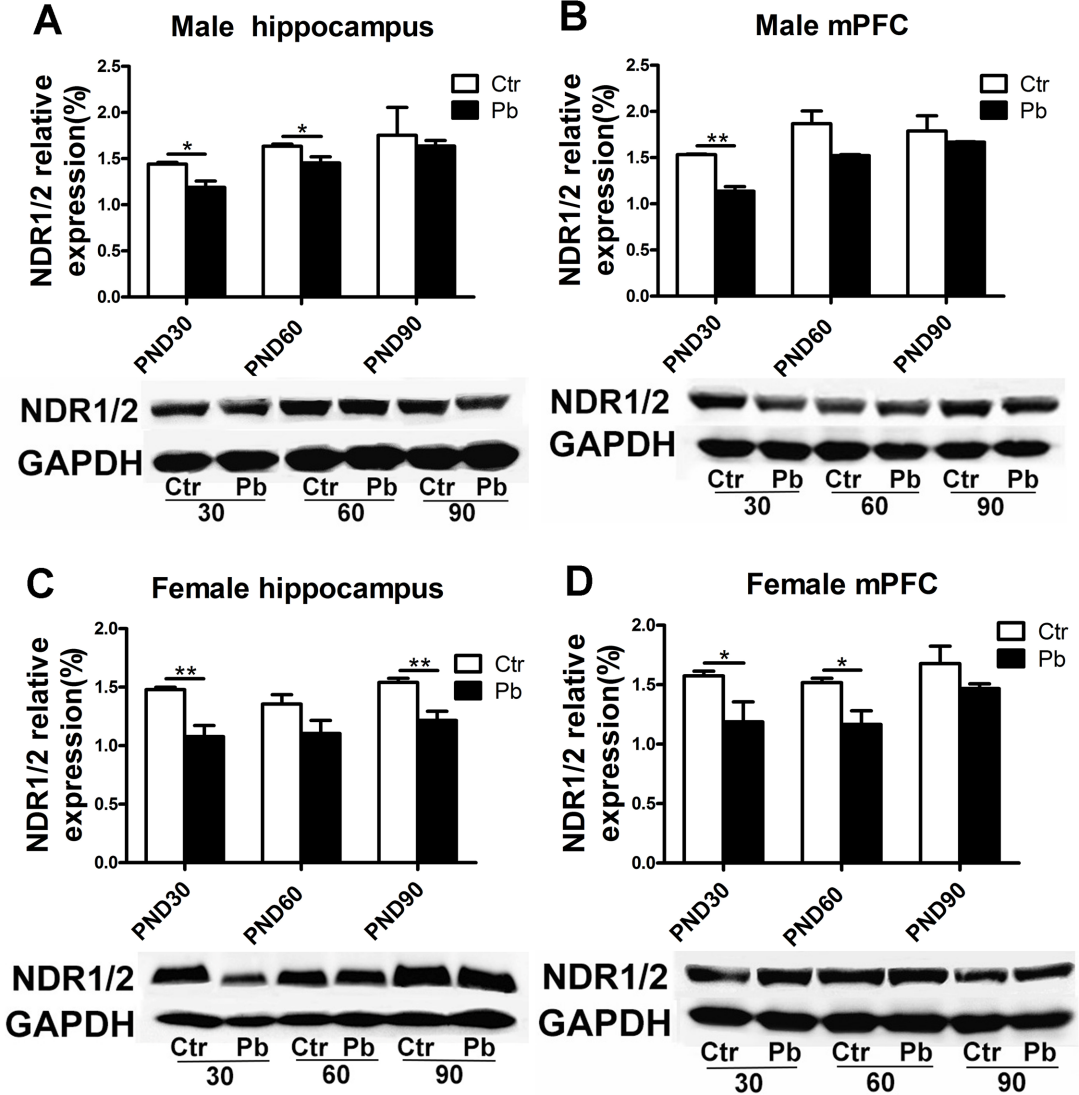
Effect of chronic Pb exposure on NDR1/2 expression in rat brain was subject to mixed factors of brain regions×age×gender. Representative Immunoblot and histograms plot showed the protein expression of NDR1/2 in male hippocampus at PND30, PND60, PND90, respectively (A), in male mPFC at PND30, PND60, PND90, respectively (B), in female hippocampus at PND30, PND60, PND90, respectively (C), in female mPFC at PND30, PND60, PND90, respectively (D). Data are expressed as mean ± SEM. *P<0.05, **P<0.01. n = 8 per group.

First we examined NDR1/2 protein expression in male rats. Pb exposure induced considerable decrease in NDR1/2 protein compared to controls at PND30 (control, 1.441±0.019; Pb exposure, 1.188±0.068, F(1,4) = 12.670, P<0.05, n = 8) and PND60 (control, 1.635±0.022; Pb exposure, 1.452±0.068, F(1,6) = 6.641, P<0.05, n = 8), except for at PND90 (control, 1.754±0.301; Pb exposure, 1.636±0.060, F(1,4) = 0.147, P>0.05, n = 8) in male hippocampus (Fig 7A). In female hippocampus, Pb exposure significantly decreased NDR1/2 expression at PND30 (control, 1.480±0.020; Pb exposure, 1.077±0.096, F(1,6) = 16.899, P<0.01, n = 8), PND90 (control, 1.541±0.035; Pb exposure, 1.215±0.080, F(1,6) = 14.000, P<0.01, n = 8), and induced marginally significant decrease at PND60 (control, 1.355±0.080; Pb exposure, 1.105±0.111, F(1,6) = 3.358, P = 0.06, n = 8) (Fig 7C). It indicated gender differences in NDR1/2 expression after Pb exposure during early adult and adulthood.

There was also a reduction in NDR1/2 protein expression in male mPFC between Pb-exposed group and control group, whereas it was considerable at PND30 (control, 1.533±0.006; Pb exposure, 1.137±0.050, F(1,4) = 61.614, P<0.01, n = 8) rather than PND60 (control, 1.869±0.136; Pb exposure, 1.522±0.011, F(1,4) = 6.449, P = 0.006, n = 8) and PND90 (control, 1.788±0.166; Pb exposure, 1.667±0.004, F(1,4) = 0.525, P>0.05, n = 8) (Fig 7B). Specifically, it indicated difference between male hippocampus and male mPFC at PND 60 (Fig 7A and 7B). Data in Fig 7D showed lower protein expression in female mPFC in Pb-exposed group than control group at all three age stages, and the reduction was notable at PND30 (control, 1.574±0.040; Pb exposure, 1.187±0.169,F(1,4) = 4.943, P<0.05, n = 8) and PND60 (control, 1.518±0.0366; Pb exposure, 1.166±0.115, F(1,4) = 8.499, P<0.05, n = 8) rather than PND90 (control, 1.677±0.148; Pb exposure, 1.468±0.040, F(1,4) = 1.871, P>0.05, n = 8) (Fig 7D). Additionally, difference was observed in mPFC at PND60 between genders (Fig 7B and 7D). Data from Fig 7C and 7D showed difference between female hippocampus and female mPFC during adulthood (early adult and adult).

In summary, chronic Pb exposure reduced NDR1/2 protein expression in rat brain. Age-dependent differences were seen in response to Pb exposure in male hippocampus, male mPFC and female mPFC. In addition, the alternation of NDR1/2 expression was also subject to gender differences.

### The alternation of *NDR1*, *NDR2* and *Rabin3* mRNA levels in rat hippocampus and mPFC following chronic Pb exposure

**Fig 8:**
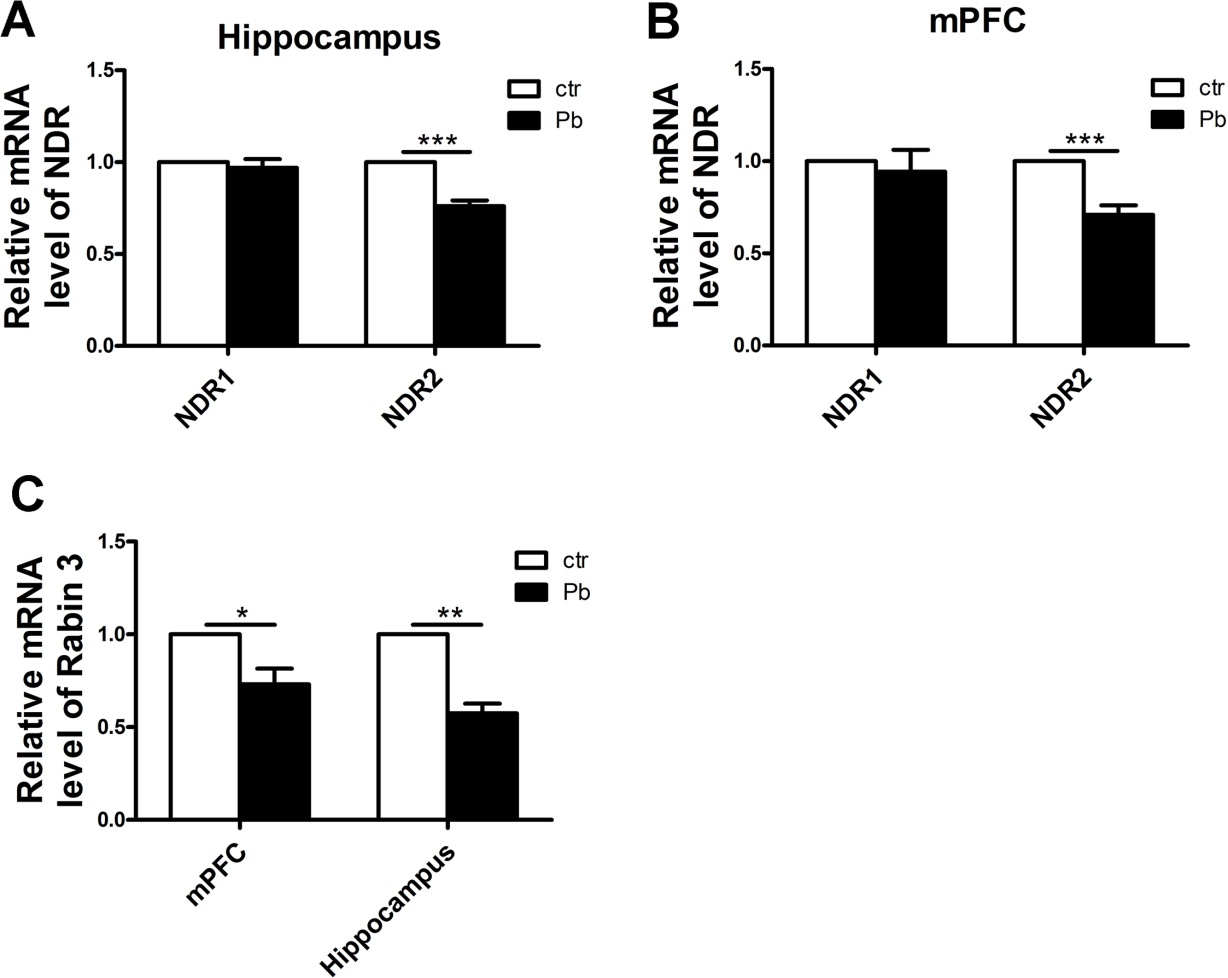
Effect of chronic Pb exposure on related key protein mRNA levels of NDR1/2 kinase pathway. *NDR1* and *NDR2* mRNA relative levels in hippocampus (A), *NDR1* and *NDR2* mRNA relative levels in mPFC (B), *Rabin3* mRNA relative levels in hippocampus and mPFC (C). The transcript amount was standardized by the amount of r-Actin in each sample. The levels of these three genes in the control group (without Pb exposure) were set as 1.0, respectively. All the results were calculated as averages of triplicate experiments. Data are expressed as mean ± SEM. *P<0.05, **P<0.01, ***P<0.001. n = 8 per group.

The above results suggested more developmental impairment followed by Pb exposure. To further explore the role of NDR1/2 kinase pathway in regulating dendrite growth by Pb exposure, we then investigated the alternation of *NDR1* and *NDR2* mRNA levels in development phase by real-time fluorescence PCR. As shown in Fig 8A, no difference was seen in *NDR1* mRNA level in hippocampus after Pb exposure (control, 1.000±0.000; Pb exposure, 0.969±0.047, F(1,13) = 0.432, P>0.05, n = 8) while the reduction in *NDR2* after Pb exposure was significant (control, 1.000±0.000; Pb exposure, 0.761±0.031, F(1,10) = 60.565, P<0.001, n = 8). A similar result was shown in Fig 8B, the decrease in *NDR1* mRNA level was not obvious (control, 1.000±0.000; Pb exposure, 0.943±0.118, F(1,20) = 0.211, P>0.05, n = 8) but in *NDR2* was considerable (control, 1.000±0.000; Pb exposure, 0.710±0.051, F(1,8) = 52.524, P<0.001, n = 8) in rat mPFC.

In view of the role of Rabin3 in regulating dendrite growth by NDR1/2 kinase, we then explored the *Rabin3* mRNA level. As data shown in Fig 8C, Pb exposure significantly decreased of *Rabin3* mRNA level in both mPFC (control, 1.000±0.000; Pb exposure, 0.731±0.09, F(1,6) = 9.954, P<0.05, n = 8) and hippocampus (control, 1.000±0.000; Pb exposure, 0.575±0.050, F(1,4) = 66.325, P<0.01, n = 8).

In summary, chronic Pb exposure decreased *NDR2* mRNA, as well as its substrate *Rabin3* mRNA in rat brain. Those results suggested the role of NDR1/2 kinase pathway in the process of regulating dendritic length and mushroom spine formation by Pb exposure.

## Discussion

As is known to all, Lead (Pb) is an important metal pollutant which results in impaired cognition and working memory function [15, 40–42]. Our recent work showed chronic Pb exposure impaired spine density [34], which we hypothesized it may be related to NDR1/2 kinase [24, 27, 29, 33]. Present study reported the impairment of dendrite growth and NDR1/2 kinase expression in response to Pb exposure with brain regions×age×gender interaction, and further the mRNA levels of key proteins of NDR1/2 kinase pathway based on those results. Our results raised four points which might be useful for further exploring Pb exposure neurotoxicity. First, Gender might play an important role in response to Pb exposure. Second, adolescence was more sensitive to chronic Pb exposure. Third, the impairment induced by Pb exposure was not parallel in hippocampus and mPFC, hippocampus was more sensitive to the neurotoxicity than mPFC. Fourth, NDR1/2 kinase expression was also affected by Pb exposure and this effect was influenced by brain regions, age or gender.

Present study first examined the alternation of dendritic length after Pb exposure. As we all know, hippocampus is necessary for memory, because of its role of conducting information processing into memory [43, 44]. Recent study showed cortical-hippocampal networks cooperatively contributed to memory [17]. Then what is the alternation of hippocampus and mPFC cognitive function in response to Pb exposure? Interestingly, although the impairment in both brain regions, hippocampal dendritic length was more sensitive to Pb exposure compared to mPFC during development (Fig 3). Dentritic length is important in synaptic transmission due to dendritic ability of receiving and transmitting information. Present study showed the hippocampal synaptic neurotoxicity caused by Pb, especially in adolescence (Fig 3).

It has been reported developmental chronic exogenous toxic exposure tended to decrease the number of mushroom spines in adult hippocampus compared to mPFC [45]. Present study found Pb exposure inhibited spine maturity in both hippocampus [18] and mPFC regardless of sex (Fig 5). In line with the experiments on animals [46, 47], this study suggested a mushroom spine explanation of impairment in long term potentiation (LTP) after Pb exposure. LTP is an important form of synaptic plasticity and a molecular basis of learning and memory [48]. Recent study demonstrated mushroom spine plays an essential role in LTP [47]. In addition, the significant inhibition induced by Pb exposure continued into adult (PND90) in hippocampus rather than mPFC (Fig 5). It indicted a more enduring impairment in hippocampal LTP compared to mPFC in response to Pb exposure. Furthermore, dendrite morphology results showed developmental phase was more sensitive to Pb exposure [15, 49] (Figs 3 and 5).

Specifically, there showed significant increase of the number of mushroom spines in mPFC at PND60 compared to PND30, as well as at PND90 compared to PND30 in Pb-exposed group (Fig 5B). It indicted that the impairment in mushroom spine formation after Pb exposure wore off from adolescence to adult in mPFC. This phenomenon also suggested developmental mPFC was much more sensitive to Pb neurotoxicity and then this toxicity became less and less. There might be several potential compensation mechanisms so that some impairment during adult tended to be reduced. As shown in Fig 3A, the impairment of hippocampal dendritic length during early adult (PND60) was parallel to that during adult (PND90), this was consistent with the findings of David et al about behavior level [15].

In considering a widespread phenomenon in brain regions: gender-dependent differences [50], present study explored whether the cognition neurotoxicity after Pb exposure is different between genders. Although no significant difference was observed in mPFC dendritic length after Pb exposure regardless sex (Fig 3B), data from Fig 4B and 4D showed difference in developmental dendritic length between genders. It suggested female was more sensitive to Pb exposure, which was consistent with Sabrina Llop et al [14, 15]. Interestingly, we failed to see significant difference in hippocampus between genders (Fig 4A and 4C). But the P value in development hippocampus in male and female was P<0.05 and P<0.001, respectively (Fig 4A and 4C). Above-mentioned more sensitivity to Pb exposure in hippocampus than mPFC might be a reasonable explanation. Results about mushroom spine also showed gender differences that Pb exposure significantly affected spine maturity from adolescence to adult in female hippocampus while the impairment was not significant in male hippocampus at PND90 (Fig 6A and 6C). It suggested a more enduring sensitivity to Pb exposure in female (from adolescence to adulthood) while the sensitivity was not significant when adult in male. It was failed to see difference in mPFC between genders (Fig 6B and 6D). These dendrite morphology results indicated that both genders suffered from Pb exposure, and a more enduring neurotoxicity was observed in female. Similar phenomenon was observed in previous studies. Girls were easier to suffer from impairment than boys following developmental Pb exposure while both genders were observed a decline in IQ [51]. Tong et al also found Pb exposure revealed a pronounced impairment in cognition in girls compared to boys [52]. As for animal study, David et al described Pb exposure affected learning and cognition, what’s more, female rats were more sensitive to this damage than male rats by behavioral experiment [15].

As we all know, protein kinase plays an important role in corresponding signaling pathway. Specifically, NDR1/2 protein kinase family was essential for dendrite growth and related signal transduction in neuronal development in mice and rats [27, 53]. Present study explored the alternation of NDR1/2 protein expression to investigate the neurotoxicity of chronic Pb exposure from translation level. Our data showed Pb exposure decreased NDR1/2 protein expression, and this alternation was significant and not influenced by gender or brain regions during adolescence (Fig 7). In addition, data showed the effect on NDR1/2 kinase expression after Pb exposure wore off from adolescence to adulthood except for female hippocampus (Fig 7). Specifically, during adulthood, there was a bit more effect in adult female hippocampus compared with adult male hippocampus (Fig 7A and 7C) and female mPFC versus to male mPFC (Fig 7B and 7D). It was reasonable to hypothesize that developmental brain was more sensitive to Pb exposure and early adult (PND60) might be a transition period (which some underlying repair mechanism occurred in this time) in response to Pb exposure. Besides, the reduction of NDR1/2 kinase expression was correlated with the impairment of spine maturity after Pb exposure (Figs 5 and 7).

To specifically explore the participation of NDR1/2 in the process of regulating dendrite growth followed by Pb exposure, present study then investigated the *NDR1* and *NDR2* mRNA levels. It was corresponding to the NDR1/2 protein expression that Pb exposure significantly decreased *NDR2* mRNA levels in both hippocampus and mPFC (Fig 8A and 8B). Thus the alternation of NDR1/2 protein expression is resulted from the regulation of *NDR1/2* mRNA levels after Pb exposure, especially *NDR2*. Furthermore, the substrate of NDR1/2, the *Rabin3* (a rat protein, which homologous to human protein Rabin8) mRNA level was significantly decreased in both hippocampus and mPFC followed by Pb exposure (Fig 8C), which was corresponding to the reduction of dendritic length and mushroom spines.

In conclusion, to our knowledge, this is the first study to explore the brain regions×age×gender effects on dendrite growth and spine maturity in response to Pb exposure. Furthermore we also provided translation and transcription evidences that the role of NDR1/2 kinase pathway in the process of regulating dendrite growth followed by Pb exposure, which may explain Pb induced dendrite morphology deficits.
